# IL35 predicts prognosis in gastric cancer and is associated with angiogenesis by altering TIMP1, PAI1 and IGFBP1

**DOI:** 10.1002/2211-5463.13005

**Published:** 2020-11-09

**Authors:** Xiao Li, Nan Niu, Jing Sun, Yiping Mou, Xujun He, Linhang Mei

**Affiliations:** ^1^ The 2nd Clinical Medical College Zhejiang Chinese Medical University Hangzhou China; ^2^ Key Laboratory of Gastroenterology of Zhejiang Province Zhejiang Provincial People’s Hospital People's Hospital of Hangzhou Medical College Hangzhou China; ^3^ Department of Gastrointestinal Surgery The Second Affiliated Hospital Wenzhou Medical University Wenzhou China; ^4^ Department of Gastrointestinal and Pancreatic Surgery Zhejiang Provincial People’s Hospital People's Hospital of Hangzhou Medical College Hangzhou China; ^5^ Department of Oncological Surgery Taizhou Hospital of Zhejiang Province Affiliated to Wenzhou Medical University Taizhou China

**Keywords:** angiogenesis, gastric cancer, IL12A, interleukin‐35, prognosis

## Abstract

Tumor angiogenesis is required for tumor growth and metastasis. Interleukin‐35 (IL35), a member of the IL12 family, is a dimer composed of IL12A and EBV‐induced gene 3（EBI3）. Elevated plasma IL35 levels have been reported to be associated with the occurrence and development of tumors. However, the role of IL35 in the angiogenesis of gastric cancer (GC) is still unclear. Here, we report that expression of IL35 is correlated with higher microvessel density, distant metastasis and poor prognosis in GC. Moreover, *in vitro* tube formation assays were performed to show that IL35 may contribute to the tube formation abilities of human umbilical vein endothelial cells. IL12A was observed to be the dominant subunit in promotion of tube formation. IL12A also inhibited expression of tissue inhibitor of metalloproteinase 1 and enhanced expression of plasminogen activator inhibitor 1 and insulin‐like growth factor‐binding protein 1 in a GC cell line. In conclusion, our data suggest that IL35 is involved in angiogenesis and is associated with poor prognosis for GC.

## Graphical abstract

Interleukin‐35 (IL35) is a dimer composed of IL12A and EBV‐induced gene 3. Here, we report that expression of IL35 is correlated with higher microvessel density, distant metastasis and poor prognosis in gastric cancer. Moreover, IL35 is involved in angiogenesis. IL12A is the dominant subunit in the promotion of tube formation.

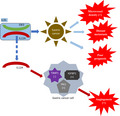

**Abbreviations**
CI: confidence interval
CM: conditioned medium
EBI3: EBV‐induced gene 3
GC: gastric cancer
HRP: horseradish peroxidase
HUVEC: human umbilical vein endothelial cell
IGFBP1: insulin‐like growth factor‐binding protein 1
IHC: immunohistochemistry
IL: interleukin
MVD: microvessel density
PAI1: plasminogen activator inhibitor 1
pc‐Ctrl: cells transfected with pcDNA3.1 empty vector
pc‐EBI3: pcDNA3.1–EBI3
pc‐IL12A: pcDNA3.1–IL12A
pYr‐Ctrl: cells transfected with pYr‐1.1 vector
rIL35: serum‐free medium with different concentrations of recombinant IL35
SD: standard deviation
sh‐EBI3: shRNA–EBI3
sh‐IL12A: shRNA–IL12A
s‐rIL35: supernatants from HGC27 cells that cultured with rIL35 for 24 h
TIMP1: tissue inhibitor of metalloproteinase‐1

Gastric cancer (GC) is currently the fifth most common malignancy in the world and the third leading cause of cancer‐related death [1]. Although rate of the age‐standardized incidence from GC has declined substantially since 1990 globally, there were still 1.2 million incident cases of GC and 865 000 deaths worldwide in 2017 [2]. Diffusion and metastasis are the main causes of death in patients with GC. When tumor cells produce specific factors, providing a more encouraging rather than suppressive environment for tumor cells, tumors become aggressive and progress toward malignancy [3]. Tumor angiogenesis is one of the important processes leading to tumor growth and metastasis [4]. When a tumor exceeds 2 mm^3^, tumor cells can secrete a variety of cytokines to promote angiogenesis [5]. Several studies have also demonstrated that antiangiogenic therapy efficiently prolongs survival in patients with advanced GC [6, 7].

Interleukin‐35 (IL35) is a new member of the IL12 family, consisting of an EBV‐induced gene 3 (Ebi3) protein and an IL12A (p35) subunit, that is primarily produced by regulatory T cells and is required for maximal immunological suppression [8]. Moreover, it is also shown to be expressed in regulatory B cells and DCs, endothelial cells, smooth muscle cells, and monocytes [9]. Recently, IL35 was investigated in various inflammatory diseases, chronic infections and cancers [10]. Importantly, the high expression of IL35 in various malignant tumor tissues and elevated plasma IL35 levels indicated the poor prognosis of tumors and was significantly associated with the occurrence and development of tumors [11, 12, 13, 14, 15, 16, 17].

Surgical removal of tumors results in a decrease in serum IL35 levels, suggesting that this cytokine can be derived from tumors [18]. Recently, tumor‐derived IL35 has emerged as a critical contributor to tumor angiogenesis. Tumor‐derived IL35 increases CD11b^+^ Gr1^+^ myeloid cell accumulation in the tumor microenvironment and promotes tumor angiogenesis [19]. Moreover, IL35 signaling promotes the angiogenesis and growth of xenograft tumors from pancreatic ductal adenocarcinoma cells in mice [20]. By increasing the expression of granulocyte‐colony stimulating factor and IL6, IL35 can up‐regulate the expression of matrix metalloproteinase‐9 and Bv8 and down‐regulate tumor necrosis factor (TNF)‐related apoptosis‐inducing ligand (TRAIL) expression in neutrophils, which enhances the proangiogenic function of neutrophils [21]. A mathematical model verified that IL35 can promote tumor growth and angiogenesis, whereas anti‐IL35 drugs can reduce tumor growth rates [22]. Furthermore, IL35 is correlated with the genesis of GC through regulating its growth and apoptosis [23]. The expressions of both EBI3 and IL12A are associated with larger tumor size and Ki‐67 expression [23]. However, the significance of tumor‐derived IL35 in GC angiogenesis and prognosis has not been clarified.

In this study, we investigated the expression of IL35 in GC tissues and its relationship with microvessel density (MVD) and prognosis. Further, *in vitro* experiments were used to explore the potential active role of IL35 in angiogenesis in GC.

## Materials and methods

### Cancer tissues and patients

A total of 106 GC paraffin‐embedded samples from January 2014 to January 2016 were collected from the Department of Gastrointestinal Surgery and Pathology of the Zhejiang Provincial People's Hospital, including various types of GC. None of the patients received radiotherapy and/or chemotherapy before surgery, nor did they receive antiangiogenic drugs after surgery. All patients were followed up for more than 5 years or until March 2019. The survival time was calculated from the date of surgery to the end of the follow‐up period and/or the date of death. The age of the patients with GC ranged from 33 to 76 years (with a median age of 58.8 years), and all cases were classified according to the *AJCC Cancer Staging Manual* (2017). This study was approved by the Ethics Committee of Zhejiang Provincial People’s Hospital with written informed consent. We confirm that the study methodologies conform to the standards set by the Declaration of Helsinki. The clinicopathological characteristics of the patients with GC are summarized in Table 1.

**Table 1:** Association between IL35 expression and clinicopathological factors. All cases were classified according to the *AJCC Cancer Staging Manual* (2017) of GC. Invasion depth (T grade) grade T1 includes T1a and T1b, and T4 includes T4a and T4b. Lymphatic metastasis (N grade) grade N3 includes N3a and N3b. TNM grade I includes Ia and Ib, TNM grade II includes IIa and IIb, and TNM grade III includes IIIa, IIIb and IIIc.

Clinical parameters	IL35 expression level			*P*
	Low (58/106)	High (48/106)	*t*/χ^2^/*Z* value	
Age, years	58.24 ± 9.91	60.12 ± 10.05	0.968	0.335
Sex, n (%)			0.020	0.889
Male	27 (56.1%)	23 (54.2%)		
Female	31 (43.9%)	25 (45.8%)		
Location, n (%)			0.632	0.729
Proximal	10 (9.1%)	7 (14.6%)		
Middle	12 (34.8%)	13 (27.1%)		
Distal	36 (56.1%)	28 (58.3%)		
Size, n (%)			2.145	0.143
≥5 cm	28 (42.4%)	30 (50.0%)		
<5 cm	30 (57.6%)	18 (50.0%)		
Histology type, n (%)			1.645	0.439
Tubular adenocarcinoma	44 (75.9%)	39 (81.2%)		
Mucinous adenocarcinoma	8 (13.8%)	3 (6.2%)		
Signet‐ring cell carcinoma	6 (10.3%)	6 (12.5%)		
Differentiation, n (%)			1.406	0.236
Well/Moderately	18 (31.0%)	10 (20.8%)		
Poorly	40 (69.0%)	38 (79.2%)		
Invasion depth (T grade), n (%)			2.289	0.515
T1	4 (6.9%)	5 (10.4%)		
T2	8 (13.8%)	3 (6.2%)		
T3	43 (74.1%)	36 (75.0%)		
T4	3 (5.2%)	4 (8.3%)		
Lymphatic metastasis (N grade), n (%)			1.197	0.754
N0	14 (24.1%)	16 (33.3%)		
N1	20 (34.5%)	15 (31.2%)		
N2	22 (37.9%)	16 (33.3%)		
N3	2 (3.4%)	1 (2.1%)		
Distant metastasis (M grade), n (%)			4.235	0.040
M0	58 (100%)	43 (89.6%)		
M1	0 (0%)	5 (10.4%)		
TNM stage, n (%)			6.508	0.089
Ⅰ	9 (15.5%)	8 (16.7%)		
Ⅱ	17 (29.3%)	12 (25.0%)		
Ⅲ	32 (55.2%)	23 (47.9%)		
Ⅳ	0 (0%)	5 (10.4%)		
Lymphatic invasion, n (%)			0.412	0.521
Yes	16 (27.6%)	16 (33.3%)		
No	42 (72.4%)	32 (66.7%)		
Vascular invasion, n (%)			1.185	0.276
Yes	42 (72.4%)	30 (62.5%)		
No	16 (27.6%)	18 (37.5%)		
Intratumoral MVD, n (%)			5.616	0.018
High	16 (27.6%)	24 (50.0%)		
Low	42 (72.4%)	24 (50.0%)		
Paracancerous MVD, n (%)			1.066	0.302
High	28 (48.3%)	28 (58.3%)		
Low	30 (51.7%)	20 (41.7%)		

### Immunohistochemistry for IL35 and MVD

For immunohistochemistry (IHC) staining, 5‐μm sections of GC tissue were used. Tissue sections were deparaffinized and rehydrated. Heat‐induced antigen retrieval was carried out in 0.01 m citrate buffer for 3 min. Sections were incubated with 3% H_2_O_2_ for 20 min at room temperature and blocked with normal goat serum. Subsequently, they were incubated with primary rabbit anti‐(human IL35) Ig (1 : 400 dilution; LAC008Hu72; Cloud‐Clone Corp., Wuhan, Hubei, China) and primary mouse anti‐(human CD105) Ig (1 : 50 dilution; MA5‐11854; Thermo Fisher Scientific, Waltham, MA, USA) overnight at 4 °C, followed by incubation with biotinylated secondary Ig at room temperature for 20 min and then with horseradish peroxidase (HRP)‐conjugated polymer (cat. no. 859043; Thermo Fisher Scientific) at room temperature for 20 min according to the manufacturer's protocol. Finally, the sections were stained with diaminobenzidine and lightly counterstained with hematoxylin for 5 min at room temperature.

IL35 was scored according to the proportion and intensity of positively stained cancer cells. For each sample, the immunoreactivity levels of IL35 were estimated under a light microscope by assessing the average signal intensity. The staining intensity was graded on a scale between 0 and 3+ (0, no staining; 1+, weak staining; 2+, moderate staining; 3+, strong staining). The percentage of cells that exhibited positive IL35 staining was scored as follows: 1 = 0–25% positive cells, 2 = 26–50% positive, 3 = 51–75% positive and 4 = 76–100% positive. The intensity and percentage scores were subsequently multiplied to obtain a composite score; a score of 0–4 was defined as low expression, whereas a score of 5–12 was defined as high expression. The degree of immunostaining was scored independently by two observers in the absence of clinical outcomes.

In this study, we used CD105 as a biomarker for MVD evaluation in GC. To evaluate MVD, we scanned sections at low magnification (×100) to identify the tumors' most prominent vasculature (hot spots). Numbers of positively stained vessels were counted in five different fields at high magnification (×400). The value of CD105 showed vessels with clearly defined lumens or well‐defined linear vessel shapes, but no single endothelial cells were defined as the MVD. All results were evaluated by two independent observers. Finally, MVD numbers greater than the average were considered the high MVD group, whereas microvessel density number less than the average were considered the low MVD group.

### Cell culture and regents

Cell lines, including MKN28, AGS, HGC27, MGC803 and GES1, were purchased from the Chinese Academy of Sciences Cell Bank of Type Culture Collection (Shanghai, China). Human umbilical vein endothelial cells (HUVECs) were purchased from ScienCell Corporation (Shanghai, China). All of the cell lines were cultured in RPMI‐1640 with 10% FBS (A1049101; HyClone, Logan, UT, USA). The cells were cultured under standard conditions with a 5% CO_2_ atmosphere at 37 °C.

### Vector construction and transfection

The IL12A (accession number: AAD16432.1) and EBI3 (accession number: AAH15364.1) constructs were generated by subcloning PCR‐amplified full‐length human cDNA into pcDNA3.1 for overexpression and were sequenced to verify correct gene insertion. They were named as pc‐IL12A (pcDNA3.1–IL12A) and pc‐EBI3, respectively. siRNA‐IL12A (CCCTTGCACTTCTGAAGAGAT) and siRNA‐EBI 3 (TGAACTGTCACTGTGAGATAT) were used for generation of shRNA vectors. The shRNA–IL12A (sh‐IL12A) and shRNA–EBI3 (sh‐EBI3) hairpin DNA sequences were annealed and synthesized as follows: IL12A forward, CACCCCCTTGCACTTCTGAAGAGATCTCGAGATCTCTTCAGAAGTGCAAGGGTTTTTTG; IL12A reverse, AGCTCAAAAAAGCCCTTGCACTTCTGAAGAGATCTCGAGATCTCTTCAGAAGTGCAAGGGC; EBI3 forward, CACCTGAACTGTCACTGTGAGATATCTCGAGATATCTCACAGTGACAGTTCATTTTTG; EBI3 reverse, AGCTCAAAAAAG TGAACTGTCACTGTGAGATATCTCGAGATATCTCACAGTGACAGTTCAC. It was cloned into pYr‐1.1 vector (Yinrun Biotechnology, Changsha, China), which was linearized by restriction enzyme BsaI. Lipofectamine 3000 (Invitrogen, Waltham, MA, USA) was used for transfection according to the manufacturer’s protocol. Stable cell lines expressing IL35 (HGC27) or shRNA–IL35 (AGS) were selected after treatment with G418 for 3–4 weeks. Cells transfected with pcDNA3.1 or pYr‐1.1 vector (containing nontargeting control shRNA sequence) were considered as negative controls and named as pc‐control [pc‐Ctrl (cells transfected with pcDNA3.1 empty vector)] and pYr‐control [pYr‐Ctrl (cells transfected with pYr‐1.1 vector)], respectively.

### Western blotting

Whole‐cell lysates of cell lines were prepared using radioimmunoprecipitation buffer (P0013J; Beyotime, Shanghai, China) according to the manufacturer's instructions. The protein concentration was determined by a bicinchoninic acid protein assay kit (23225; Thermo Fisher, Rockford, IL, USA). Subsequently, 40 μg of proteins was used for SDS/PAGE and transferred to poly(vinylidene difluoride) membranes. The membranes were blocked for 2 h in 5% nonfat dry milk in TBST and then incubated with the primary antibodies overnight at 4 °C. After incubation with the secondary HRP‐coupled antibodies for 2 h at room temperature, the membranes were washed with TBST, and the immunosignal was developed with enhanced chemiluminescence reagent and exposed in a ChemiDoc XRS + System (1708265; Bio‐Rad, Hercules, CA, USA). β‐Actin was used as the internal control. The concentrations and sources of primary antibodies are as follows: tissue inhibitor of metalloproteinase 1 (TIMP1; ab109125; Abcam, Cambridge, MA, USA) was 1 : 1000, plasminogen activator inhibitor 1 (PAI1; ab222754; Abcam) was 1 : 800, insulin‐like growth factor‐binding protein 1 (IGFBP1; ab181141; Abcam) was 1 : 1000, IL35 (LAC008Hu72; Cloud‐Clone Corp.) was 1 : 1000 and β‐actin (AF5001; Beyotime) was 1 : 2000.

### Real‐time quantitative RT‐PCR analysis

The total RNA was extracted from cells. Then 1 μg RNA was reverse transcribed to cDNA using a PrimeScript™ RT Master Mix (Perfect Real Time) Kit (RR036A; Takara, Shiga, Japan) according to the manufacturer's instructions. GAPDH was used as the internal control. We used SYBR® Green–based real‐time PCR assays with the following primers: EBI3 forward, 5′‐TCATTGCCACGTACAGGCTC‐3′, EBI3 reverse, 5′‐GGGTCGGGCTTGATGATGTG‐3′, IL12A forward, 5′‐CCTTGCACTTCTGAAGAGATTGA‐3′, IL12A reverse, 5′‐ACAGGGCCATCATAAAAGAGGT‐3′, TIMP1 forward, 5′‐CTTCTGCAATTCCGACCTCGT‐3′, TIMP1 reverse, 5′‐ACGCTGGTATAAGGTGGTCTG‐3′, PAI1 forward, 5′‐ACCGCAACGTGGTTTTCTCA‐3′, PAI1 reverse, 5′‐TTGAATCCCATAGCTGCTTGAAT‐3′, IGFBP1 forward, 5′‐TTGGGACGCCATCAGTACCTA‐3′, IGFBP1 reverse, 5′‐TTGGCTAAACTCTCTACGACTCT‐3′, GAPDH forward, 5′‐TTGCAACCGGGAAGGAAATG‐3′ and GAPDH reverse, 5′‐TGGAATTTGCCATGGGTGGA‐3′. The PCR parameters were as follows: 95 °C for 4 min, followed by 40 cycles of 95 °C for 10 s, 60 °C for 30 s and 72 °C for 30 s. After PCR, melting curve analysis was performed. The relative expression levels were compared with the expression level of GAPDH and were calculated using the $2^{‐\Delta \Delta C_{t}}$ method.

### Preparation of conditioned media

The GC cells were seeded in a T75 tissue culture flask and grown to 40–50% confluence (depending on the growth rate of the cell lines). The growth medium was then replaced with serum‐free medium to further culture for 24 h, and the supernatants were harvested for future assays. For neutralization experiments, the neutralizing antibody against TIMP1, PAI1 or IGFBP1 was preincubated at 4 °C with 1 mL supernatant for 6 h, then used for HUVECs coculture in tube formation experiments. The neutralizing antibodies against TIMP1 (AF970‐SP; R&D Systems, Minneapolis, MN, USA), PAI1 (AF1786‐SP; R&D Systems), IGFBP1 (MAB675‐SP; R&D Systems) and control antibody (MAB003; R&D Systems) were 0.2, 1.5, 20 and 5 µg·mL^−1^, respectively.

The conditioned media (CMs) used in the experiments were: (a) rIL35, serum‐free medium with different concentrations of recombinant IL35 (Cloud‐Clone Corp., RPC008Hu01); (b) supernatants from HGC27 cells that were cultured with rIL35 for 24 h; (c) supernatants from the pc‐IL12A, pc‐EBI3 and pc‐Ctrl HGC27 cells, respectively; (d) supernatants from the sh‐IL12A, sh‐EBI3 and pYr‐ctrl AGS cells, respectively; (e) supernatants from pc‐IL12A HGC27 cells pretreated with PAI1, IGFBP1 or control neutralizing antibodies for 6 h, respectively; and (f) supernatants from sh‐IL12A AGS cells pretreated with TIMP1 or control neutralizing antibodies for 6 h, respectively.

### Tube formation assay

HUVECs (8 × 10^3^) were then cultured with 150 µL CM in 96‐well plates precoated with 50 µL of growth factor–deprived Matrigel. After incubating at 37 °C for 3 h, the images were taken under 100× microscope. Then the sum of the length of the trees composed from segments and branches in the analyzed area (total branching length) were analyzed by using image j software (National Institutes of Health, Bethesda, MD, USA). The unit of length is pixel.

### Profiling of angiogenesis‐related proteins

To explore the angiogenesis‐related proteins that were induced by IL12A and EBI3 overexpression in GC cells, we used a proteome profiler human angiogenesis array kit (ARY007; R&D Systems) for screening, and the processes were according to the manufacturer's protocol. In brief, the pc‐IL12A, pc‐EBI3 and pc‐Ctrl HGC27 cells were collected for total protein extraction, and a total of 500 µg protein per sample was applied to the respective array membrane. After incubation with HRP‐labeled detection antibody, an enhanced chemiluminescence kit was used, and the chemiluminescence signal was exposed and collected by ChemiDoc System (Bio‐Rad). Positive signals were quantified using image lab 6.0 software (Bio‐Rad, Hercules, CA, USA). The mean signal of duplicate spots representing each angiogenesis‐related protein was normalized to the mean value of the reference signals present on the array.

### Statistical analysis

Statistical analyses were performed by using spss 20.0 software (IBM, Armonk, NY, USA). The relationship between IL35 and clinicopathological characteristics was tested using the chi‐square test or Wilcoxon rank‐sum test. Each experiment was performed in triplicate, and the values are presented as the mean ± standard deviation (SD). The variance between the groups was statistically compared. Student's *t*‐test or one‐way ANOVA with the Dunnett *post hoc* test was used to compare mean values. Survival curves were plotted using the Kaplan–Meier method and were compared by log rank test. The significance of various survival‐related variables was assessed by a Cox regression model in a multivariate analysis. A *P* value < 0.05 was defined as statistically significant.

## Results

### Correlation between IL35 expression and clinical variables

To investigate the expression pattern of IL35 in GC tissues, we used immunohistochemical analysis to detect IL35 in 106 GC samples. Based on the IL35 immunoreactivity scores, 48 (45.3%) of 106 GCs were considered high expression. Patients with high IL35 expression had a higher distant metastasis rate (10.4% or 5/48) than patients with low IL35 expression (0% or 0/58; *P* < 0.05). The proportion of high intratumoral MVD in patients with high IL35 expression (50.0% or 24/48) was greater than that in patients with low IL35 expression (27.6% or 16/58; *P* < 0.05). No other variables, such as age, sex, tumor diameter and degree of differentiation, were correlated with IL35 expression (Table 1).

### Correlation between MVD and clinical variables

**Fig. 1:**
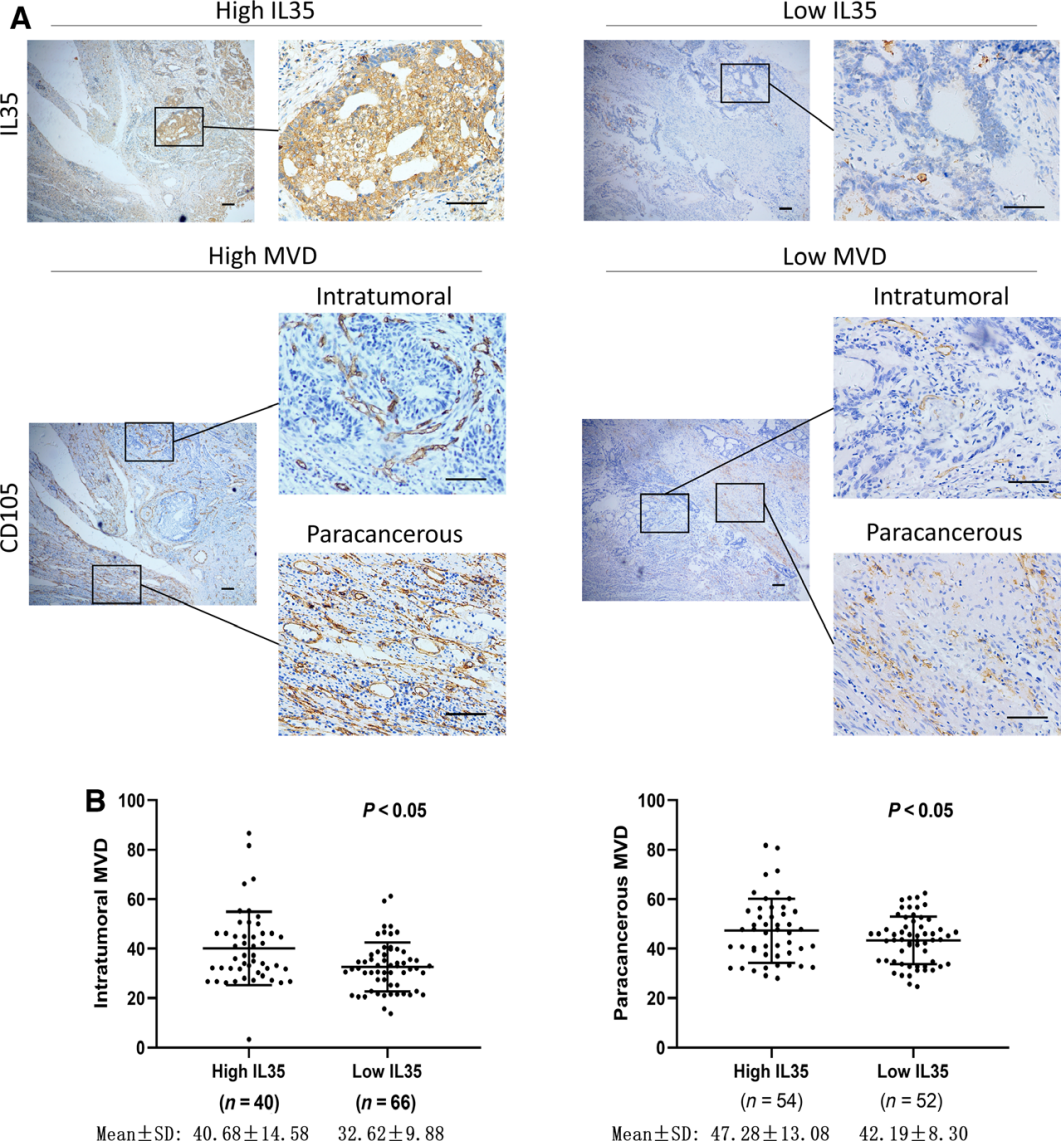
IL35 increases MVD in GC tissues. (A) Representative images of IHC staining for IL35 and CD105 in human GC tissues are shown. (B) Quantification of MVD with different IL35 levels is presented. Shown as the mean ± SD.Scale bars, 100 μm.

In this cohort of patients (*n* = 106), the mean numbers of intratumoral and paracancerous MVDs were 36.27 ± 18.58 and 45.17 ± 11.57, respectively. Intratumoral MVD in the poorly differentiated group (38.28 ± 14.01) was significantly higher than that in the well‐differentiated and moderately differentiated groups (30.68 ± 5.92; *P* < 0.05), and a similar result was observed for paracancerous MVD (46.90 ± 12.00 versus 40.35 ± 8.78; *P* < 0.05) (Table 2). In addition, the patients with high expression of IL35 had a higher intratumoral MVD than those with low IL35 expression (40.68 ± 14.58 versus 32.62 ± 9.88; *P* < 0.05). Similar to the intratumoral MVD, the paracancerous MVD in high‐IL35‐expressing patients was also higher than that in low‐IL35‐expressing patients (47.28 ± 13.08 versus 42.19 ± 8.30; *P* < 0.05) (Table 2 and Fig. 1).

**Table 2:** Association between MVD number and clinicopathological factors. All cases were classified according to the *AJCC Cancer Staging Manual* (2017) of GC. Invasion depth (T grade) grade T1 includes T1a and T1b, and T4 includes T4a and T4b. Lymphatic metastasis (N grade) grade N3 includes N3a and N3b. TNM grade I includes Ia and Ib, TNM grade II includes IIa and IIb, and TNM grade III includes IIIa, IIIb and IIIc.

Clinical parameters	Intratumoral MVD number (mean ± SD)	*t*/χ^2^ or *F* value	*P*	Paracancerous MVD number (mean ± SD)	*t*/χ^2^ or *F* value	*P*
Sex		0.389	0.698		1.148	0.254
Male	36.66 ± 11.62			46.19 ± 11.88		
Female	35.65 ± 14.54			43.54 ± 11.01		
Location		2.247	0.111		1.948	0.148
Proximal	28.43 ± 8.34			39.53 ± 7.35		
Middle	36.14 ± 10.82			43.92 ± 11.08		
Distal	37.56 ± 13.96			46.67 ± 12.12		
Size		0.235	0.814		0.312	0.756
≥5 cm	36.57 ± 11.37			45.53 ± 10.86		
<5 cm	35.98 ± 14.17			44.82 ± 12.31		
Histology type		0.431	0.731		0.074	0.974
Tubular adenocarcinoma	36.63 ± 13.94			45.35 ± 12.28		
Mucinous adenocarcinoma	31.96 ± 6.82			45.38 ± 8.50		
Signet‐ring cell carcinoma	37.33 ± 7.31			43.67 ± 9.51		
Differentiation		2.775	0.007		2.643	0.009
Well/Moderately	30.68 ± 5.92			40.35 ± 8.78		
Poorly	38.28 ± 14.01			46.90 ± 12.00		
Invasion depth (T grade)		2.872	0.412		1.978	0.577
T1	34.37 ± 5.74			43.43 ± 5.91		
T2	39.07 ± 14.38			53.60 ± 19.56		
T3	35.20 ± 10.96			44.05 ± 9.88		
T4	31.17 ± 0.93			51.33 ± 21.55		
Lymphatic metastasis (N grade)		0.702	0.553		1.804	0.151
N0	38.80 ± 15.79			47.59 ± 12.86		
N1	36.41 ± 11.89			41.64 ± 11.04		
N2	33.93 ± 8.86			43.62 ± 10.53		
N3	35.49 ± 13.47			47.64 ± 10.91		
Distant metastasis (M grade)		0.281	0.779		1.091	0.278
M0	36.36 ± 13.14			44.87 ± 11.23		
M1	34.83 ± 5.72			50.17 ± 16.79		
TNM stage		0.651	0.885		1.669	0.644
Ⅰ	39.65 ± 18.72			46.96 ± 15.03		
Ⅱ	35.25 ± 10.38			45.60 ± 9.69		
Ⅲ	35.36 ± 12.45			45.20 ± 10.47		
Ⅳ	36.69 ± 7.68			42.58 ± 13.19		
Lymphatic invasion		0.515	0.607		0.486	0.628
Yes	35.85 ± 11.21			42.11 ± 21.14		
No	37.25 ± 16.10			46.00 ± 10.78		
Vascular invasion		0.294	0.769		0.370	0.712
Yes	35.74 ± 12.93			45.77 ± 11.08		
No	36.52 ± 12.85			44.88 ± 11.86		
IL35 expression		3.261	0.002		2.276	0.025
High	40.68 ± 14.58			47.28 ± 13.08		
Low	32.62 ± 9.88			42.19 ± 8.30		

Other clinical variables, such as invasion depth, lymphatic metastasis, lymphatic invasion, histology type, distant metastasis and TNM stage, were also included in the analysis of microvessel density number between each group (Table 2).

### High expression of IL35 and increased MVD in GC tissue predict poor prognosis

**Fig. 2:**
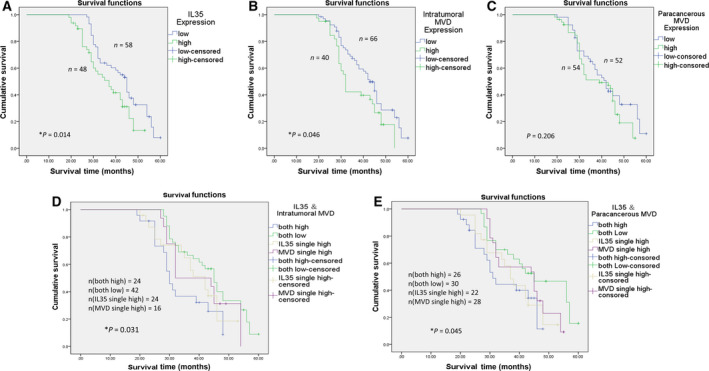
Kaplan–Meier survival curves of patients with GC. (A) Survival curve of patients with low IL35 and high IL35 expression. (B) Survival curve of patients with low and high intratumoral MVD. (C) Survival curve of patients with low and high paracancerous MVD. (D) Survival curve of the correlation between IL35 and intratumoral MVD. (E) Survival curve of the correlation between IL35 and paracancerous MVD. *P < 0.05.

In this cohort of patients (*n* = 106), the median survival time was 40.51 months [95% confidence interval (CI): 38.14–42.87 months]. The survival time of the high IL35 expression group was significantly shorter than that of the low IL35 expression group [36.58 months (95% CI: 33.39–39.76 months) versus 43.10 months (95% CI: 40.12–46.08 months); *P* < 0.05; Fig. 2A]. The survival time of patients with high intratumoral MVD was 36.86 months (95% CI: 33.26–40.46 months), which was significantly shorter than that of patients with low expression [42.40 months (95% CI: 33.26–40.46 months); *P* < 0.05; Fig. 2B]. The survival time of patients with high paracancerous MVD was also slightly shorter than that of patients with low expression [38.82 months (95% CI: 35.77–41.86 months) versus 42.35 months (95% CI: 38.84–45.85 months)], but no significant difference was found (*P* = 0.206; Fig. 2C). Moreover, Kaplan–Meier analysis indicated significantly worse survival in patients with both high IL35 and high MVD. The survival time of patients with both high IL35 and high intratumoral MVD [33.98 months (95% CI: 29.78–40.51 months)] was shorter than that of patients with single IL35‐high [38.73 months (95% CI: 34.30–43.16 months)], single MVD‐high [40.31 months (95% CI: 34.80–45.82 months)] or double low ones [43.96 months (95% CI: 40.51–47.41 months)] (*P* < 0.05; Fig. 2D). Similarly, patients with both strong IL35 expression and high paracancerous MVD also exhibited a significantly shorter mean survival time [34.77 months (95% CI: 30.66–38.88 months)] than patients with single IL35‐high [37.86 months (95% CI: 33.55–42.16 months)], single MVD‐high [41.43 months (95% CI: 37.82–45.05 months)] or double low ones [45.16 months (95% CI: 40.51–49.82 months)] (*P* < 0.05; Fig. 2E). A Cox multivariate analysis indicated that size, differentiation, invasion depth, lymphatic metastasis and IL35 expression were independent risk factors in this GC cohort (Table 3).

**Table 3:** Multivariate analysis as determined by Cox regression analysis in 106 patients with GC. HR, hazard ratio.

Clinicopathological parameters	*B*	SE	Wald	*P*	HR	95.0% CI for HR
Size	0.883	0.264	11.179	0.001	2.418	1.441–4.056
Differentiation	0.824	0.340	5.868	0.015	2.279	1.170–4.437
Invasion depth	1.049	0.365	8.243	0.004	2.856	1.395–5.845
Lymphatic metastasis	0.453	0.168	7.286	0.007	1.573	1.132–2.186
IL35 expression	0.742	0.287	6.676	0.010	2.101	1.196–3.689

### IL35 facilitated the tube formation of HUVECs in an indirect manner

**Fig. 3:**
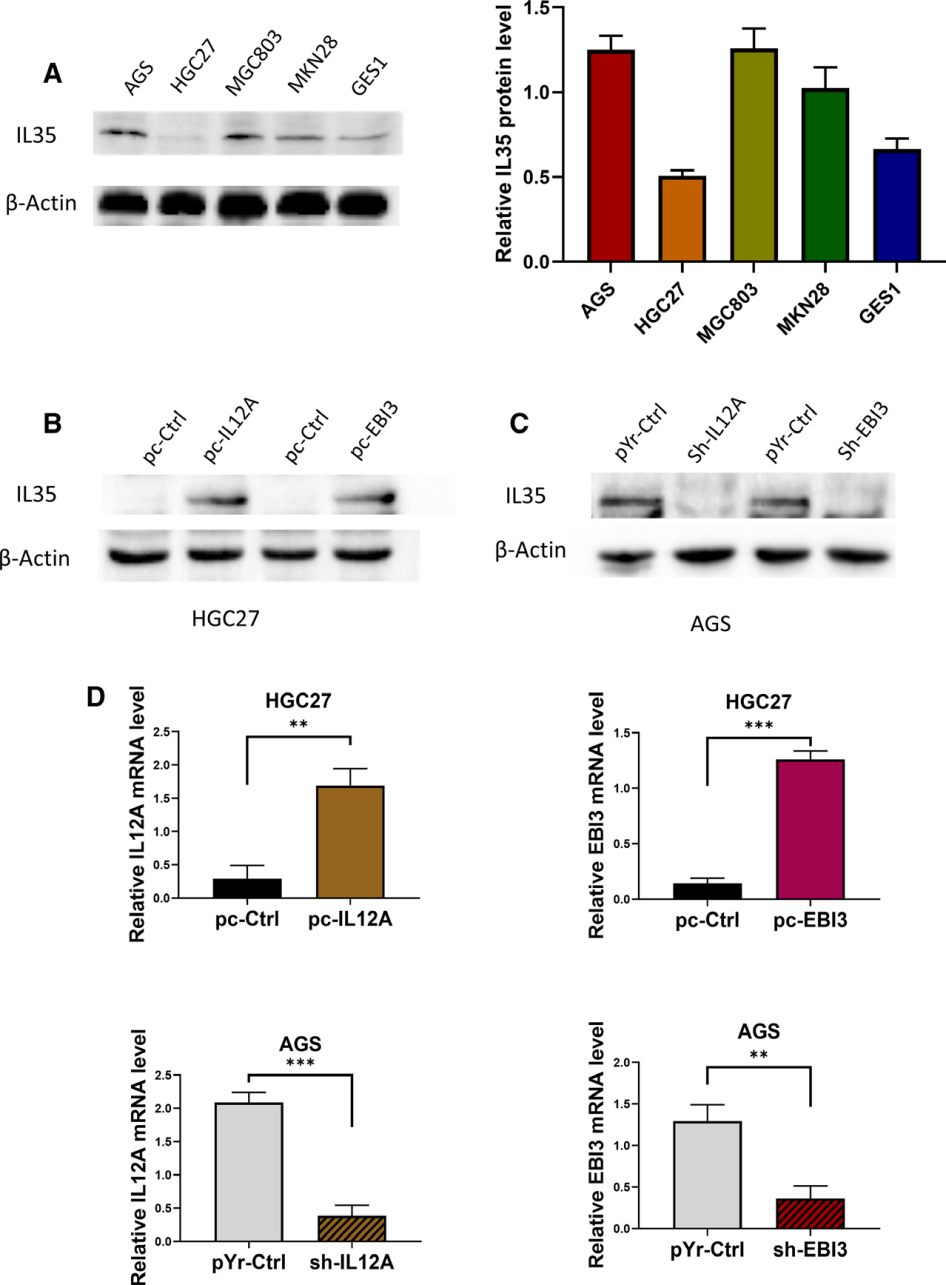
Construction of IL35‐overexpressing HGC27 cell lines and IL35‐knockdown AGS cell lines. (A) Western blot analysis of IL35 in each cell line, including AGS, HGC27, MGC803, MKN28 and GES1. (B) Western blot analysis of IL35 in HGC27 cells transfected with pc‐IL12A, pc‐EBI3 and pc‐Ctrl vectors. (C) Western blot analysis of IL35 in AGS cells transfected with sh‐IL12A, sh‐EBI3 and pYr‐Ctrl vectors. (D) The expressions of IL12A and EBI3 in indicated HGC27 and AGS cells were verified by real‐time quantitative PCR. Shown as the mean ± SD; statistical test: Student’s*t*‐test;*n* = 3;**P < 0.01, ***P < 0.001.

To investigate the potential mechanism of promoting angiogenesis of IL35, we first measured the expression of IL35 in several commonly used GC cell lines and normal gastric epithelial cells, including AGS, HGC27, MGC803, MKN28 and GES1. We found that the HGC27 and AGS cell lines had a relatively low and high expression of IL35 among GC cells, respectively. Thus, HGC27 cell lines were selected for transfecting with pc‐IL12A and pc‐EBI3 vectors to overexpress IL35 and were verified by western blot and real‐time quantitative PCR assays. Meanwhile, AGS cell lines were transfected with sh‐IL12A and sh‐EBI3 vectors to knock down the IL35 expression and were verified by western blot and real‐time quantitative PCR assays (Fig. 3).

**Fig. 4:**
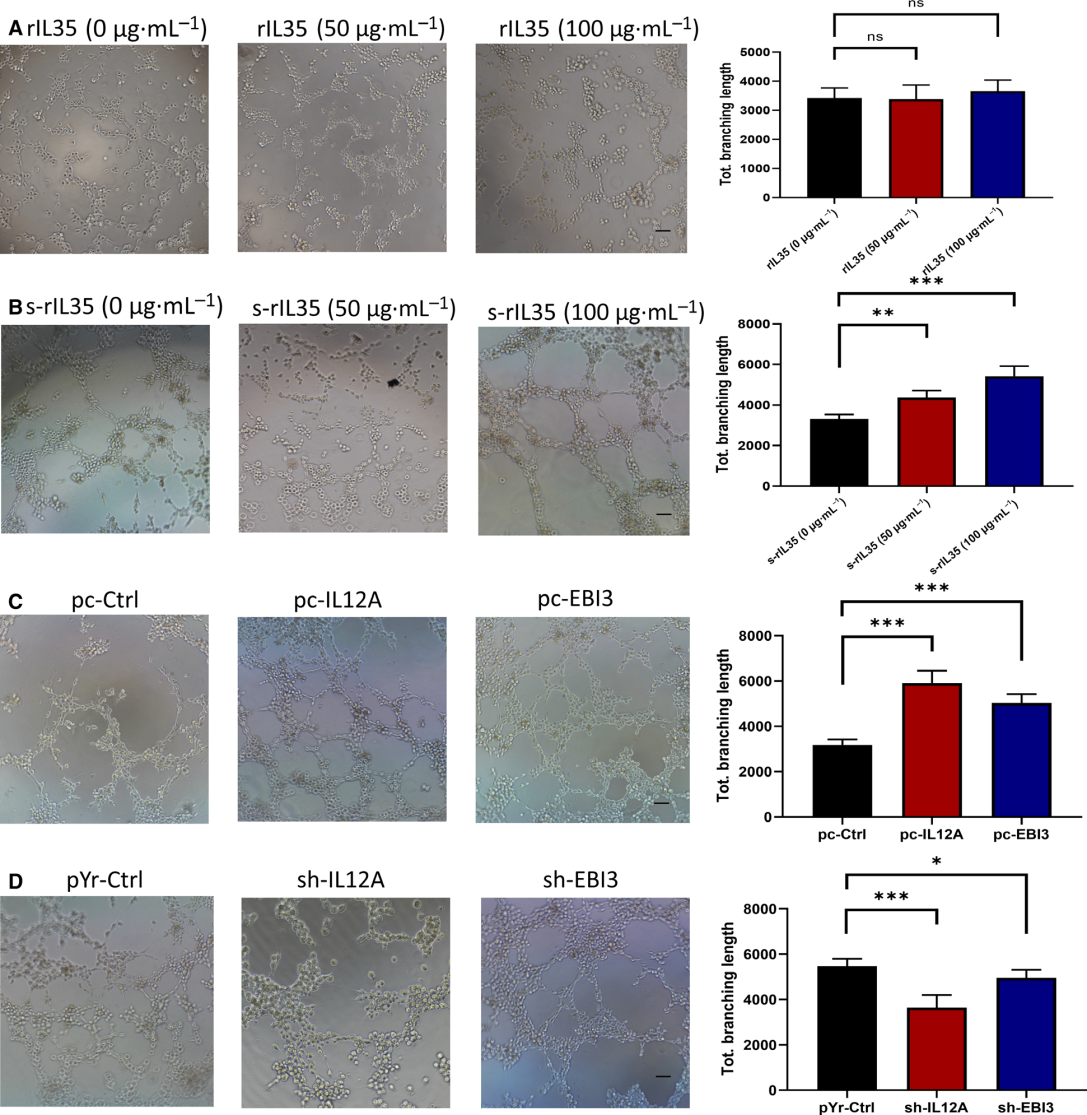
IL35 facilitates the tube formation of HUVECs in an indirect manner. HUVECs suspended in CM were seeded into Matrigel‐coated 96‐well plates. Sum of length of the trees composed from segments and branches in the analyzed area (Tot. branching length) were analyzed by usingimage jsoftware. The unit of length is pixel. (A) Serum‐free RPMI‐1640 medium with different concentrations of recombinant IL35 were used as the CM (rIL35). (B) Supernatants from HGC27 cells that cultured with rIL35 for 24 h were used as the CM [supernatants from HGC27 cells that cultured with rIL35 for 24 h (s‐rIL35)]. (C) Supernatants from the pc‐IL12A, pc‐EBI3 and pc‐Ctrl HGC27 cells were used as the CM. (D) Supernatants from the sh‐IL12A, sh‐EBI3 and pYr‐ctrl AGS cells were used as the CM. Data are shown as the mean ± SD; statistical test: one‐way ANOVA with the Dunnett*post hoc*test;*n* = 3; *P < 0.05, **P < 0.01, ***P < 0.001. Scale bars, 300 μm. ns, no statistical difference.

Then, we performed the *in vitro* tube formation assay to explore the angiogenesis effects of IL35 expression by directly treating HUVEC cells with rIL35. Unexpectedly, they had no significant effect on tube formation of HUVECs, which stimulated directly with rIL35 (0, 50 and 100 µg·mL^−1^) (Fig. 4A). However, as shown in Fig. 4B, after the HGC27 cells were cultured with different concentrations of rIL35 medium for 24 h, the supernatants were collected and used as CM for tube formation assays, which resulted in a substantial increase of tube formation. Similarly, the supernatants from pc‐IL12A and pc‐EBI3 HGC27 cells had a significant promotion effect in HUVECs tube formation. Also, it was found that the IL12A subunit had a stronger ability to promote tube formation than EBI3 (Fig. 4C). At the same time, the supernatants CM from pYr‐IL12A and pYr‐EBI3 AGS cell were also used in the tube formation assay, and it was found that the number of tube formations was decreased compared with corresponding controls (Fig. 4D). Therefore, we hypothesized that IL35 indirectly participated in tumor angiogenesis by regulating other angiogenic factors from tumor cells.

### IL35 facilitates angiogenesis by regulating TIMP1, PAI1 and IGFBP1 *in vitro*

**Fig. 5:**
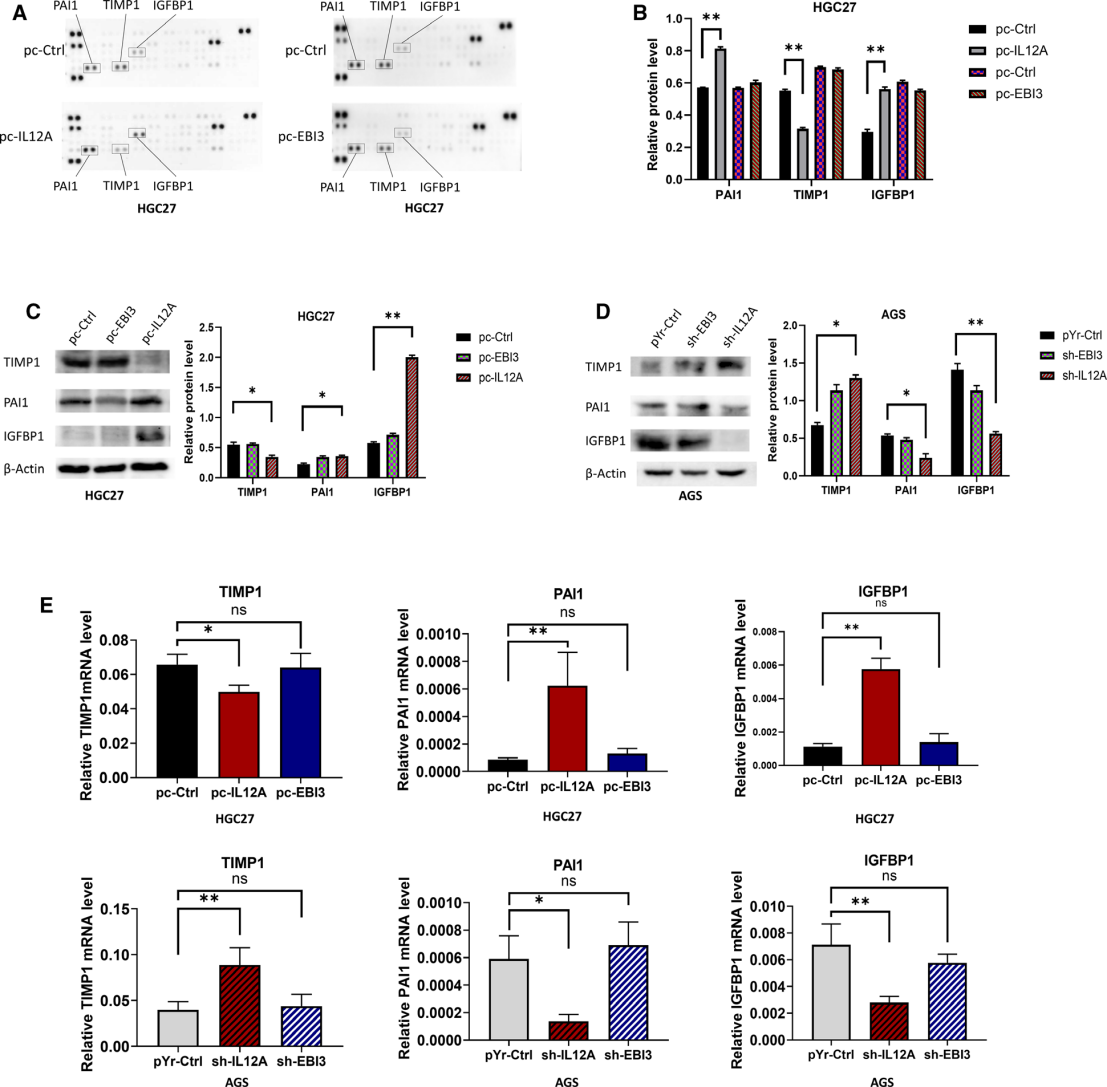
IL12A regulates expression of angiogenesis‐related proteins in GC cells, including TIMP1, IGFBP1 and PAI1. (A, B) Angiogenesis‐related proteins of indicated HGC27 cells were detected with proteome profiler human angiogenesis array kit. (C) Relative expressions of TIMP1, PAI1 and IGFBP1 were detected by western blotting in pc‐IL12A, pc‐EBI3 and pc‐Ctrl HGC27 cells. (D) Relative expressions of TIMP1, PAI1 and IGFBP1 were detected by western blotting in sh‐IL12A, sh‐EBI3 and pYr‐ctrl AGS cells. (E) TIMP1, PAI1 and IGFBP1 were detected by real‐time quantitative PCR in indicated HGC27 and AGS cells. Data are shown as the mean ± SD; statistical test: Student’s*t*‐test and one‐way ANOVA with the Dunnett*post hoc*test;*n* = 3; *P < 0.05, **P < 0.01. ns, no statistical difference.

To explore the potential molecules that are involved in IL35 angiogenesis promotion, we chose a proteome profiler human angiogenesis array to determine the potential factors that may be involved in IL35 angiogenesis. Interestingly, we found that the expression of the angiogenic growth‐related factor TIMP1 was decreased, whereas PAI1 and IGFBP1 were increased with the overexpressed IL12A in HGC27 cells. However, there were no significant changes in the factors in the HGC27 cells transfected with EBI3 (Fig. 5A,B). These results indicate that the IL12A subunit of IL35 may be the major subunit for angiogenesis promotion.

For verifying these results, we conducted western blotting and real‐time quantitative PCR to assess the levels of TIMP1, PAI1 and IGFBP1 in HGC27 cells with IL35 overexpression and AGS cells with IL35 knockdown (Fig. 5C–E). The results were consistent with proteome profiler human angiogenesis array; that is, the up‐regulation of IL12A resulted in the decrease of TIMP1 and the increase of PAI1 and IGFBP1.

**Fig. 6:**
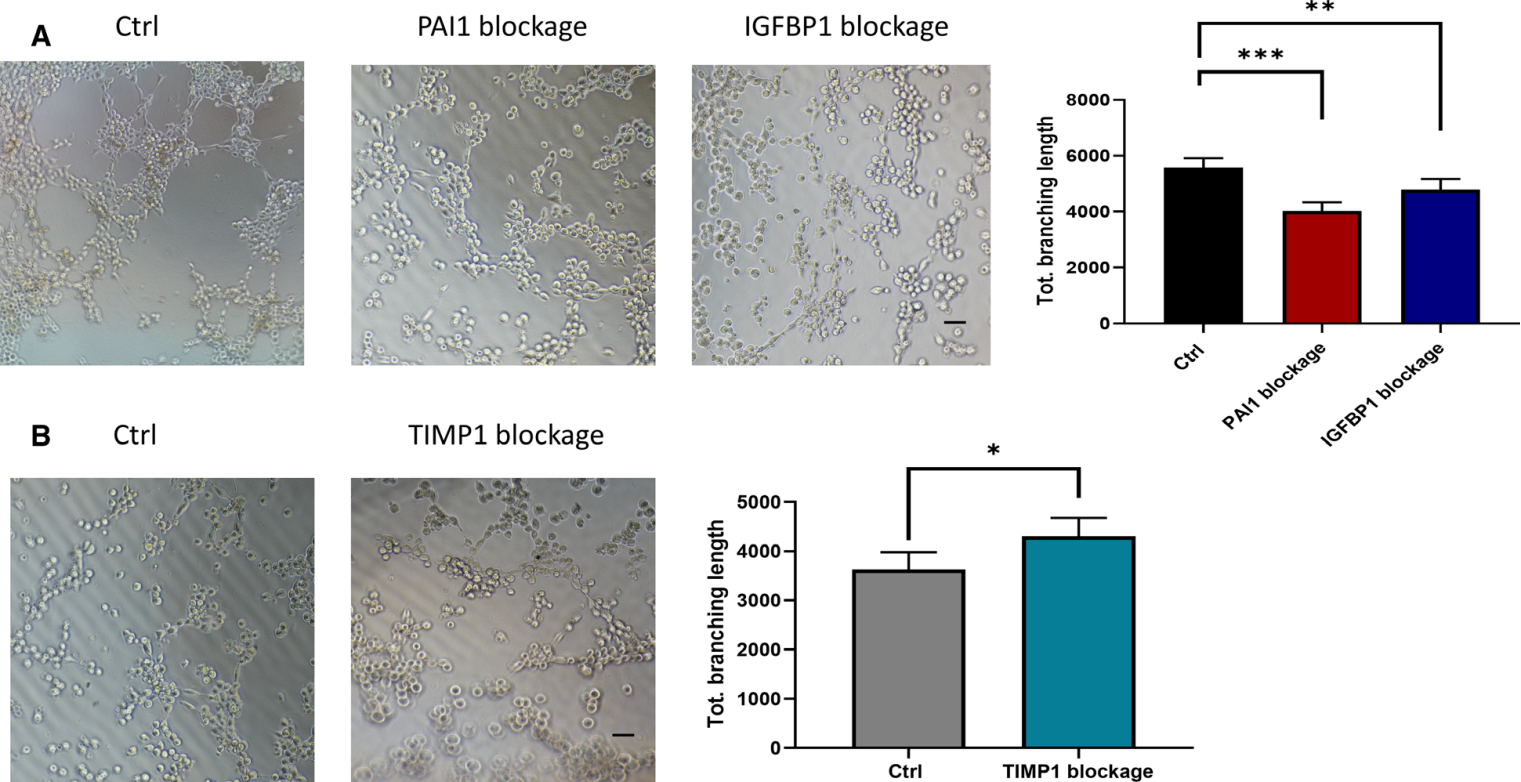
Blocking TIMP1, PAI1 and IGFBP1 affects IL12a‐mediated tube formation. HUVECs suspended in CM were seeded into Matrigel‐coated 96‐well plates. Sum of length of the trees composed from segments and branches in the analyzed area (Tot. branching length) were analyzed by usingimage jsoftware. The unit of length is pixel. (A) Supernatants from pc‐IL12A HGC27 cells pretreated with PAI1, IGFBP1 or control neutralizing antibodies (Ctrl) for 6 h were used as the CM. (B) Supernatants from sh‐IL12A AGS cells pretreated with TIMP1 or control neutralizing antibodies (Ctrl) for 6 h were used as the CM. Data are shown as the mean ± SD; statistical test: Student’s*t*‐test and one‐way ANOVA with the Dunnett*post hoc*test;*n* = 3; *P < 0.05, **P < 0.01, ***P < 0.001. Scale bars, 300 μm.

To further demonstrate that the proangiogenesis role of IL12A is achieved by regulation of TIMP1, PAI1 and IGFBP1, we used neutralizing antibodies to block TIMP1, PAI1 and IGFBP1 in the CMs (supernatants from sh‐IL12A AGS cells and pc‐IL12A HGC27 cells, respectively) and performed tube formation experiments again. As shown in Fig. 6A, blocking PAI1 and IGFBP1 in supernatants from pc‐IL12A HGC27 cell resulted in a significant decrease in tube formation. However, anti‐TIMP1‐neutralizing serum effectively promoted the tube formation activity of supernatants from sh‐IL12A AGS cells (Fig. 6B).

## Discussion

Accumulating evidence has shown that dysregulated neovascularization is involved in GC tumor proliferation and metastasis [24, 25]. MVD represents the degree of angiogenesis, which is also significantly correlated with aggressive behaviors and poor prognosis [24]. Although the vascular endothelial growth factor pathway is critical for tumor blood supply and growth, tumor angiogenesis is a complex process that may involve the use of pathways other than vascular endothelial growth factor [26, 27]. Therefore, the further understanding of proangiogenic pathways in GC would be beneficial for developing novel antiangiogenic drugs. In this study, we demonstrated that high expression of IL35 is associated with angiogenesis via regulation of TIMP1, PAI1 and IGFBP1 in patients with GC and predicts poor prognosis, including higher rates of distant metastasis and shorter survival time.

As a novel molecule, IL35 plays an important role in alleviating inflammation‐related tissue damage and promoting tumor progression by inducing a potent regulatory T cell population [28]. In addition, tumor‐derived IL35 could increase the expression of IL35 through positive feedback, which further promotes tumor immune escape and is conducive to tumor progression [19, 29]. In GC, EBI3 and IL12A expression are strongly related to larger tumor size [23]. Furthermore, EBI3 expression is correlated with invasion depth in GC [23]. These results suggest that IL35 might be involved in the growth of GC. However, the relationship between IL35 and MVD has not yet been reported in GC. Based on our IHC assay, we found that high expression of IL35 was detected in 45.3% (48/106) of GC specimens, which was positively associated with high MVD in GC nests, and similar spatial adjacencies have been reported in pancreatic ductal adenocarcinoma [20]. In addition, we also found that patients with both high IL35 expression and high MVD in tumors had the poorest prognosis compared with the single high or double low group. Also, a Cox multivariate analysis indicated that a high level of IL35 was an independent risk factor in the patients with GC. The other noteworthy finding was that patients with high IL35 expression had significantly higher rates of distant metastasis. Other studies have reported that increased expressions of IL35 [30] and MVD [31] in tumor adjacent tissues are significantly associated with tumor metastasis. These data support that IL35 may be involved in tumor angiogenesis and distant metastasis, which leads to a poor prognosis of GC.

In addition, we investigated the potential proangiogenic mechanism of IL35 and observed that IL35 facilitated angiogenesis *in vitro* by regulating TIMP1, PAI1 and IGFBP1. Blocking TIMP1 has been shown to promote angiogenesis in many studies [32, 33, 34]. In GC, the inhibition of TIMP1 also increased angiogenesis *in vivo* [35]. Also, PAI1 knockdown in GC cells reduced angiogenesis *in vitro*, suggesting that tumor cell‐derived PAI1 is also critical for GC angiogenesis [36]. Moreover, silencing IGFBP1 expression in microglial cells or neutralizing it with antibodies can reduce the ability of cells to induce angiogenesis, diminishing its involvement in tumor angiogenesis [37]. IGFBP1 can be regulated by lysophosphatidic acid, which enhances the angiogenic capability of human chondrocytes by regulating Gi/nuclear factor‐κB‐dependent angiogenic factor expression [38]. Based on the earlier experimental results and other study results, we propose that the IL12A subunit of IL35 is a major promoter of angiogenesis that regulates TIMP1, PAI1 and IGFBP1 expression.

Several potential limitations of this study should be noted. First, IL35 is composed of two subunits, EBI3 and IL12A. However, because of the restriction of the antibody recognition site, in our IHC study, the IL35 antibody could not distinguish these two different subunits of IL35, leading us to have no way of knowing which subunit was more dominant in promoting tumor angiogenesis. Fortunately, to address this, we constructed overexpression and knockdown cell lines of IL12A and EBI3 and observed that IL12A may be more dominant in angiogenesis. Second, we found a correlation between IL35 and distant metastasis of GC, the specific mechanism of which has not been revealed. Further investigations, such as studies regarding signaling pathways, are warranted to obtain an understanding of the role of IL35 in GC metastasis.

## Conclusions

This study demonstrates that IL35 is highly expressed in GC and could be a potential biomarker for the prognosis of GC. More importantly, IL35 is correlated with the MVD of GC and mediates angiogenesis by regulating TIMP1, PAI1 and IGFBP1. This molecule is a promising candidate for the development of new antiangiogenic drugs.

## Conflict of interest

The authors declare no conflict of interest.

## Author contributions

XL and XH conceived the study and designed the experiments. XL, NN and JS performed the experiments and carried out the analysis. XL drafted the manuscript. LM analyzed data and revised the manuscript. YM was responsible for the supervision of the project and approved the final version of the manuscript. All of the authors have contributed to and approved the manuscript.

## Data Availability

The datasets used and analyzed during this study are available from the corresponding author on reasonable request.
